# Knowledge and Attitude Towards the Elderly Among Doctors and Medical Students: A Questionnaire-Based Study

**DOI:** 10.7759/cureus.56732

**Published:** 2024-03-22

**Authors:** Abdullah Al Ghailani, Abdullah Al Lawati, Fatma Al Kharusi, Ammar Al Shabibi, Anas Al Wahaibi, Ali Al Wardi, Abdullah Alyafai, Hamed Al Sinawi

**Affiliations:** 1 Psychiatry and Behavioral Sciences, Sultan Qaboos University Hospital, Muscat, OMN; 2 Behavioral Medicine, College of Medicine and Health Sciences, Sultan Qaboos University, Muscat, OMN; 3 Emergency Medicine, Sultan Qaboos University Hospital, Muscat, OMN

**Keywords:** doctors, medical student, knowledge attitude, elderly people, geriatric psychiatry

## Abstract

Introduction: There is a continuous rise in the total number and percentage of elders globally, and as such, they are expected to utilize healthcare services more often. Therefore, this study aimed to determine doctors' and students' current knowledge and attitudes toward elders and compare those findings with other studies worldwide. The specific objectives of this study were to determine and compare the differences in attitudes between medical students and doctors regarding geriatrics. This comparison will focus on the following four key domains: social values, resource distribution, compassion, and medical care. Another objective was to assess the knowledge of medical students and doctors regarding geriatric topics. This assessment will help determine the necessity for interventions such as educational programs and workshops on geriatrics.

Methods: This cross-sectional questionnaire-based study was conducted by disseminating a Google Forms survey to medical students and doctors. The survey included the University of California, Los Angeles (UCLA) Geriatrics Attitudes Scale and the UCLA Geriatrics Knowledge Test. Data was analyzed using SPSS version 29.0.2.0 (Armonk, NY: IBM Corp.).

Results: A total number of 126 medical students and 72 doctors filled out the survey. Both medical students and doctors demonstrated moderate scores on the attitudes scale, with overall average scores of 2.92 out of 5 and 2.93 out of 5, respectively. As for knowledge, medical students achieved an average score of 41%, while doctors attained an average score of 43%.

Conclusion: This study provides significant insights regarding the knowledge and attitudes of students and doctors and attitudes towards geriatrics. The moderate attitudes score and poor knowledge score across both groups indicate the need for medical educators in Oman to further emphasize and teach about geriatrics in medical curricula.

## Introduction

As of 2020, an estimated one billion people worldwide are over 60 years of age. This number is predicted to rise to 1.4 billion in 2030, with people aged 80 years or over expected to reach 426 million [1]. In Oman, the elderly constitute only 2.5% of the total population. However, this number is expected to rise to 13% (or around 888,000 people) by 2050 [2]. Elders are generally considered to be frail and cause a burden to society as some of them develop common health conditions, such as diabetes, hypertension, hearing loss, cataracts, bone pain, and osteoarthritis. Moreover, as they age, they may develop some common mental health conditions such as depression and dementia [3]. As such, the elderly population will need to access health services more often, and thus, doctors and medical students will be more likely to encounter and treat them [4]. Furthermore, elders need some essential behavior, knowledge, and skills to be taken care of. Thus, it is important to encourage positive attitudes among doctors and medical students towards the care of the elderly to ensure better medical care [5]. Therefore, it is essential to determine the current knowledge and attitudes of doctors and medical students in Oman towards elderly care to determine possible areas for improvement.

Numerous studies worldwide and in the region have explored this topic. A study in Saudi Arabia found that medical and nursing students had moderate knowledge and attitudes towards elderly care. Nursing students had higher knowledge scores, while medical students showed better attitude scores [6]. Another study conducted in Malaysia also found medical students to have moderate knowledge about the elderly. However, it showed a statistically significant association between knowledge and attitude towards the elderly and the presence of grandparents, area of residence, and number of siblings [7].

Therefore, given the increased likelihood of utilization of healthcare services by the elderly, as well as the expected rise in the percentage of elderly in the overall population, it is of utmost importance that doctors and medical students have good knowledge and positive attitudes towards elderly care. As such, we aimed to investigate how medical students and practicing doctors currently perceive older people and to measure and analyze the extent of their knowledge about this matter. In Oman, a limited number of studies address issues related to the geriatric population. Therefore, understanding the attitude toward older people and its predictor of sociodemographic character will play a significant role in determining the social need for educational programs to help spread awareness when it comes to elder abuse. This study's outcomes will help design teaching modules and courses on elderly care among students and doctors. In addition, the findings will shed light on the current situation, which could lead to increased awareness of elderly care among healthcare workers and the population in general.

## Materials and methods

Study design and sample size

This cross-sectional questionnaire-based study used the University of California, Los Angeles (UCLA) Geriatrics Attitudes Scale and the UCLA Geriatrics Knowledge Test. Both scales are validated and publicly available. In addition, studies that have utilized those scales indicated high reliability, with the attitude scale having a Cronbach's alpha value of 0.76 and the knowledge scale 0.71 [8,9]. Participants in this study comprised the following two groups: medical students and doctors. A convenience sampling method was employed to recruit 126 medical students and 72 doctors of different professional levels.

Inclusion and exclusion criteria

All doctors practicing in Oman, regardless of their professional level, were considered eligible for inclusion in the sample, including recent graduates. Regarding students, all Sultan Qaboos University (SQU) medical students were considered for inclusion, except for those studying foundation courses, as they have not yet been exposed to the medical curriculum. Nursing and biomedical sciences students were excluded from the study as they did not align with this study's objectives.

Questionnaire and data collection

Data collection was conducted through a survey created in Google Forms. Participants were given clear instructions on completing the questionnaires and knowledge tests. Confidentiality and anonymity of responses were ensured throughout the data collection process. The questionnaires used are described below.

Attitudes toward geriatrics

Attitudes toward geriatrics were assessed using the 14-item UCLA Geriatrics Attitudes Scale. However, one item about paying for healthcare was deemed not applicable to our population, as the majority of our geriatric population receives free healthcare from the government. Therefore, we omitted this item, resulting in a modified 13-item scale. The scale is a widely recognized instrument for capturing attitudes toward older individuals and has been previously validated in various healthcare settings [10]. This modification allowed for a more contextually relevant assessment of attitudes within our specific population. Each item was rated on a 5-point Likert scale, ranging from strongly agree (5) to strongly disagree (1), with a neutral response marked as 3. This adapted scale aimed to measure participants' attitudes toward geriatrics comprehensively.

Knowledge in geriatrics

The participants' knowledge of geriatrics was evaluated using the 23-item UCLA Geriatrics Knowledge Test with minor changes in wording to conform to local use of terms and updated content to relate to recent data related to geriatric medicine. The knowledge test comprised 15 multiple-choice questions and eight factual/false statements. Questions covered various topics, including social and ethical issues, geriatric syndromes, and general internal medicine knowledge application to older patients. Participants' scores were reported as the total number of correct responses.

Statistical analysis

Quantitative data from the geriatric attitude questionnaire and knowledge test were analyzed using SPSS version 29.0.2.0 (Armonk, NY: IBM Corp.). Descriptive statistics, including means, standard deviations, and percentages, were calculated to summarize participants' attitudes and knowledge of geriatrics. Comparative analyses, such as t-tests or ANOVA, will be performed to assess differences between medical students and doctors.

## Results

The sample comprised a cohort of 126 medical students and 72 doctors in Oman, offering a comprehensive exploration of the attitudes and knowledge in geriatric care across different healthcare professionals. Among the medical student participants, 69.0% (n=88) are female, while 30.2% (n=38) are male, indicating a predominant representation of female participants. For the doctor cohort, 68.1% (n=49) were female, and 31.8% (n=23) were male, reflecting a similar trend of a higher proportion of female participants. The demographic data of both students and doctors is shown in Table 1.

**Table 1:** Demographic data of the sample including doctors and medical students.

Gender	Number	Percentage
Students (n=126)		
Female	88	69.8%
Male	38	30.2%
Total	126	100.0%
Doctors (n=72)		
Female	49	68.1%
Male	23	31.9%
Total	72	100.0%

Additionally, the doctors were subclassified based on their professional levels, revealing insights into the distribution of roles within healthcare. A detailed breakdown of roles is presented in Table 2 below, providing a nuanced understanding of the diverse perspectives contributed by doctors of different professional backgrounds.

**Table 2:** Doctors’ roles in the healthcare field comprising the sample.

Role	Number	Percentage
Consultant	5	6.9%
Senior specialist	2	2.8%
Specialist	21	29.2%
Resident/medical officer	37	51.4%
Intern	7	9.7%
Total	72	100.0%

**Figure 1:**
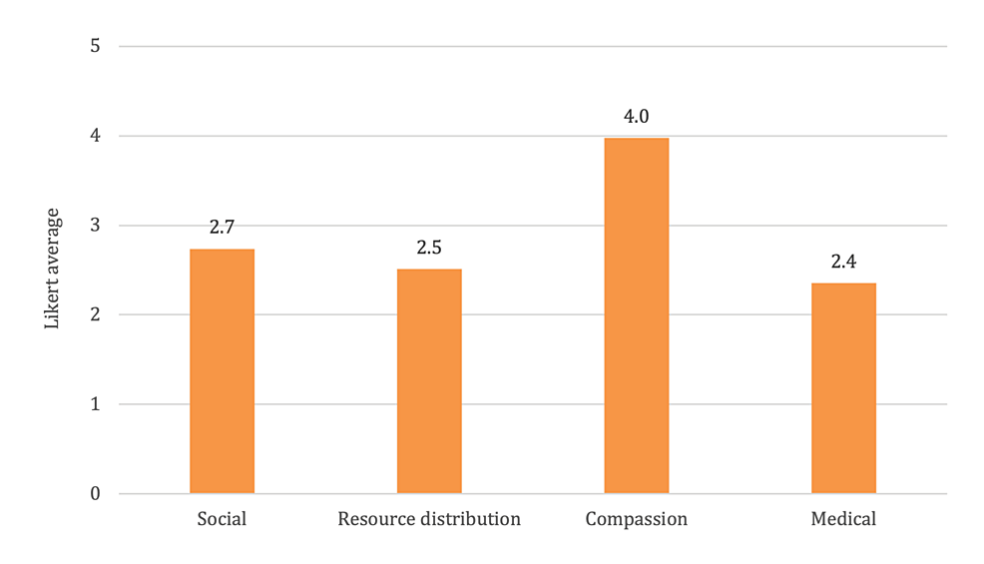
Medical students’ attitudes towards geriatrics.

An evaluation of attitudes toward the elderly using the UCLA Geriatric Attitude Scale revealed distinct patterns among medical students and doctors. As shown in Figure 1, medical students demonstrated generally positive attitudes with mean scores of 2.7 in social values, 2.5 in resource distribution, 4.0 in compassion, and 2.4 in medical care, with an average of 2.93 out of 5 across all domains. Notably, the lower score in the medical care sub-division suggests potential hesitancy or age-related biases among this cohort in providing medical care for the elderly.

**Figure 2:**
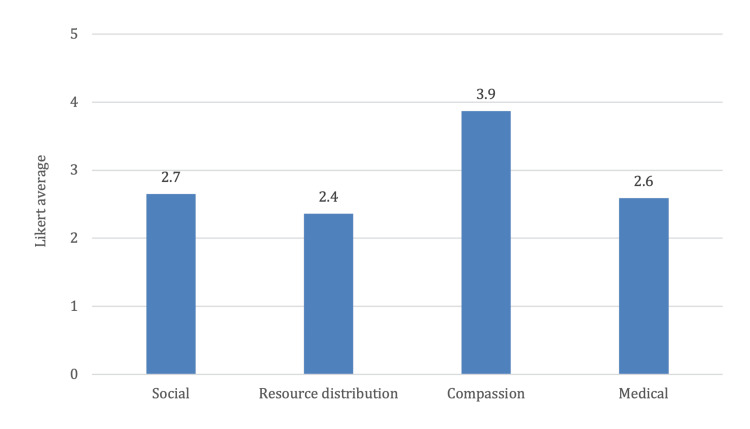
Doctors’ attitudes towards geriatrics.

**Figure 3:**
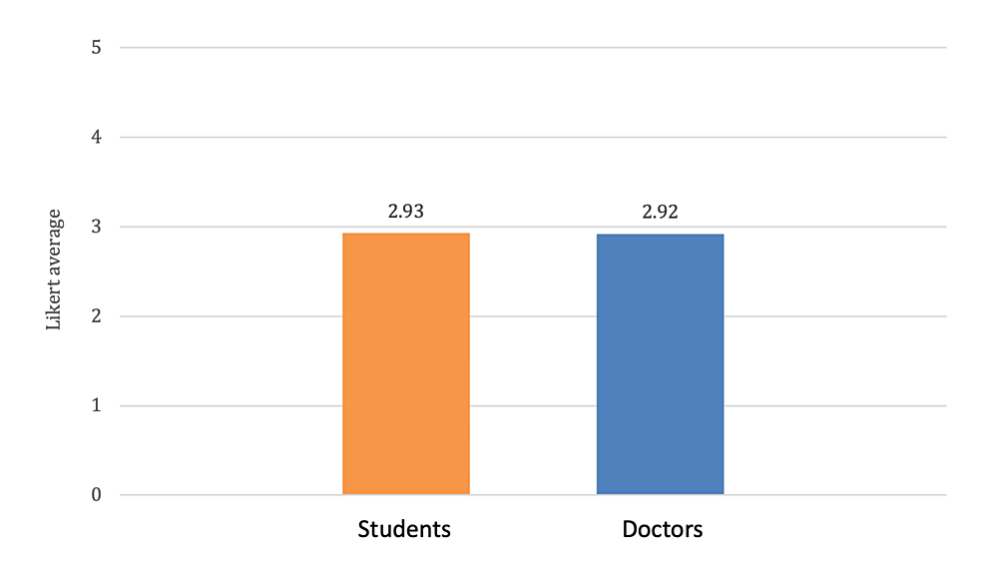
Five-domain average scale of attitudes towards geriatrics.

For doctors, as shown in Figure 2, the results showcased a varied perspective with mean scores of 2.7 in social values, 2.4 in resource distribution, 3.9 in compassion, and 2.6 in medical care, with an average of 2.92 across all domains. As shown in Figure 3, both groups had similar scores in terms of the overall average score across the five domains. Exploring these attitudes within the medical community is crucial to understanding potential barriers and facilitators to effective geriatric care.

**Figure 4:**
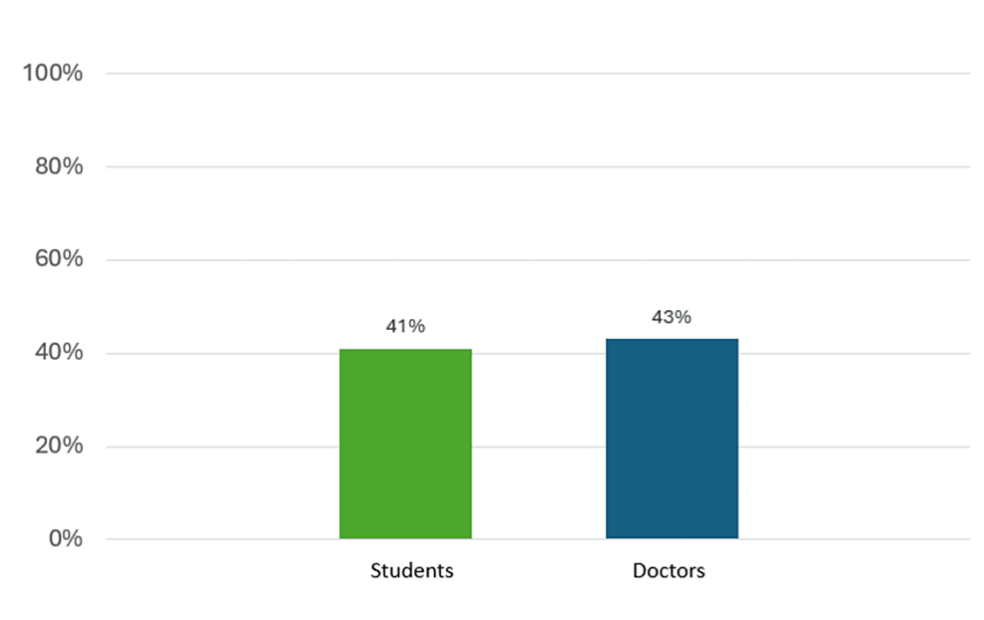
Overall knowledge of geriatrics among students and doctors.

The participants' scores were calculated as the percentage of correct responses. As shown in Figure 4, medical students achieved an average score of 41%, while doctors attained an average score of 43%. This modest difference suggests a relatively comparable level of knowledge between the two groups. Statistical analyses were calculated to summarize participants' knowledge scores, including descriptive statistics, means, and standard deviations. An independent samples t-test was also performed to compare the mean knowledge scores between medical students and doctors. The t-test results revealed no significant difference in knowledge scores between the two groups (p>0.05), indicating a similar level of knowledge in geriatrics. Despite the lack of a significant difference, it is notable that the overall knowledge level demonstrated by medical students and doctors was found to be relatively low and that there is room for improvement in understanding geriatric topics among these healthcare cohorts. These findings underscore the importance of targeted educational interventions and training programs to enhance knowledge and promote a more informed approach to geriatric care.

## Discussion

Our study revealed intriguing insights into medical students' and doctors' knowledge levels and attitudes toward geriatric care. The similarity in knowledge scores between these two groups, as indicated by the non-significant difference in the t-test, may suggest a shared foundational understanding of geriatric topics. However, it is essential to acknowledge that the overall knowledge demonstrated by both cohorts was relatively low, consistent with findings in the literature [11]. Regarding attitudes towards the elderly, the average score across all four domains in our study was 2.93 out of 5 for medical students and 2.92 out of 5 for doctors. This is lower than the 3.37 out of 5 scores among medical and nursing students in Saudi Arabia [6]. A study conducted in Malaysia that used the Geriatric Attitude Scale also concluded that its sample had moderate knowledge and attitudes regarding elderly care [7]. In 2009, two published studies explored the attitudes towards the elderly among medical students and junior doctors in Singapore. The result showed a positive attitude toward the elderly by 98.2% and 99.2% of first- and fourth-year medical students, respectively [4].

Our sample's observed low knowledge levels (41% medical students, 43% doctors) underscore the pressing need for targeted interventions in geriatric education within medical curricula. Several studies have highlighted the need for more emphasis on geriatric topics in medical training programs, contributing to suboptimal care for older patients worldwide [12,13]. The fact that medical students and doctors exhibited similarly low levels of knowledge suggests that improvements are needed at various stages of medical education and practice.

In our study, a noteworthy 19.6% (n=27) of participants within the medical student group expressed the belief that older individuals do not contribute to society. This finding indicates that a certain proportion of medical students hold a perception that may contribute to ageist attitudes. Understanding and addressing such attitudes is crucial in healthcare education, as fostering positive perceptions of aging can affect the quality of care provided by future healthcare professionals. Further research into the variables influencing these attitudes and creating focused treatments would be necessary to encourage a more comprehensive and age-inclusive approach in medical education. Moreover, our results reflect the importance of cultivating positive attitudes toward geriatric care. Although not directly assessed for their impact on clinical practice, the attitudes captured in our study may influence the quality of care provided to older adults. Previous research has indicated that negative attitudes among healthcare professionals can lead to disparities in care and impact patient outcomes [14,15].

This study has some limitations that future studies should attempt to minimize or address. Firstly, there is a potential bias among participants filling out the form, given that the questionnaire used in the study is self-administered. Another limitation is that this is a single-center study, and thus the findings cannot be generalized to all medical students and doctors in Oman. Finally, the questionnaire used was entirely in English, which might be a barrier to the participants' complete understanding, as the majority of them are native Arabic speakers, and English is considered a second language. Future studies should be mindful of these limitations and strive to avoid them.

## Conclusions

This study highlights that medical students and doctors in the sample possess moderate attitudes and poor knowledge regarding elderly care, indicating a need for greater geriatrics education in the medical curriculum and more geriatrics courses and workshops for doctors. Doctors and students are compassionate towards the elderly, and thus, the focus should be on the other domains they lack. Further studies with more extensive samples and across different institutions are needed to make the results generalizable in Oman.
